# Monitoring of Measuring Devices Using a Programmable Logic Controller and a Dedicated Desktop Application

**DOI:** 10.3390/s22239313

**Published:** 2022-11-30

**Authors:** Bartosz Kwapisz, Michał Doligalski, Marek Ochowiak, Marcin Mrugalski, Sylwia Włodarczak, Andżelika Krupińska, Magdalena Matuszak, Szymon Woziwodzki

**Affiliations:** 1 Department of Chemical Engineering and Equipment, Poznan University of Technology, 60-965 Poznan, Poland; 2 Institute of Control and Computational Engineering, University of Zielona Góra, Licealna 9, 65-417 Zielona Góra, Poland

**Keywords:** PLC, algorithm, flow rate, measurement, control, logic controller

## Abstract

An appropriate control and measurement system is one of the elements that allows for the safe and effective operation of a technological installation. Such a system may have a diverse structure that corresponds to the expectations and needs of its user. The devices of various manufacturers, including PLC (programmable logic controllers), can be used as a source of measurement data. This enables flexible measurement–control installations, which are adapted to the technological installations, to be built. This paper presents a system, which was created by the authors, for monitoring the temperature, pressure and flow rate of fluids. It uses the operational and IT properties of the PLC and is adapted to an existing installation on the test stand for multiphase spraying processes. In addition, as a part of the research, an application written in Python language, which allows collected data to be displayed, stored and exported, was implemented.

## 1. Introduction

In order to control every chemical process, it is necessary to know the parameters that are required with regards to the engineer’s point of view [1]. Only then can it be known what product is obtained, whether there is unnecessary waste of energy or raw materials, and whether it meets the desired properties [2,3]. Therefore, it is important to have appropriate measuring equipment that will facilitate and accelerate the examination of the condition of the process. The older methods of collecting measurements, which use manual devices operated by trained personnel, are being abandoned. Automated systems, due to technological development, are more often used. They ensure accurate and fast results on both a laboratory and industrial scale, while at the same time, replacing humans in these tasks. The latest technological achievements provide opportunities to limit any unforeseen human impact on the process and to reduce the risk of measurement errors. In subsequent steps, they lead to full automation, which is only controlled by a human. This allows for an increase in the efficiency, and the improvement in the quality of the products, while at the same time reducing the consumption of the substrates and utilities [4,5,6].

### 1.1. Measuring Transducers

Measuring transducers are separate sets of basic elements, thanks to which it is possible to convert an input measured quantity (such as length, mass or temperature) or an already preprocessed measured quantity into an outgoing quantity with a changed value or value with a changed size. These kinds of transducers are made of three types of elementary parts [7,8]: those that cause losses of energy dissipated into heat, those that store kinetic energy and those that store potential energy.

The measurement system is a combination of such transducers, and it creates a chain of receiving and processing input signals. When considering the above-mentioned carrier waves, i.e., the energy waves, the transducers are devices that process this energy. Therefore, in the measurement systems, it is possible to systematize the transducers with regards to the type of energy [8,9,10,11]: mechanical, chemical, magnetic, electric, optical, thermal, acoustic, and nuclear decay.

The signal processing in an ideal transducer can be mathematically modeled, and it is expressed in the following operations [8,12]:

- the tracking operation: the input signal is accurately mapped at the output, taking into account the amplification factor, which is expressed as a constant. Such processing has no time delay. Mathematically, it can be expressed by the following equation:

(1) y(t)=k∗x(t)

- the delay operation: the output signal is delayed by a constant value when compared to the input signal:

(2) $$y\left(t\right)=k*x\left(t-t_{0}\right)$$

- the averaging operation: for a certain fixed period of time, which is called the averaging time *t_u_*, the value of the output signal is determined, and it results from the average of the input signal:

(3) $$y\left(t\right)=\frac{k}{t_{u}}\int_{t_{u}}^{t}x\left(\tau \right)d\tau$$

For dynamic measurements, the results can be averaged in two ways: first, for periodic signals, the averaging time *t_u_* can be assumed to be equal to period *T* for the measured phenomenon, and second, for non-periodic signals, the averaging time *t_u_* should be selected with regards to the nature of the measurements, their interpretation, etc.

- the integration operation: the output signal is integrated with the constant *t_c_*, integration time. The following dependency is used:

(4) $$\frac{t_{c}*dy\left(t\right)}{dt}=k*x\left(t\right)$$

The finite boundaries are the times of the measurement duration. It is important that the constant component of the signals is equal to 0, otherwise there will be a constantly increasing output signal to be dealt with.

- the differentiation operation: the output signal is a derivative of the input signal:

(5) $$y\left(t\right)=\frac{k*t_{r}*dx\left(t\right)}{dt}$$

where the constant *t_r_* is the differentiation time constant.

- the filtering operation: the specific harmonic components are removed from the frequency spectrum of the signal, i.e., specific bands or band ranges. A distinction is made between a low-pass filtering (cutting off the frequency from the bottom to specific values), a high-pass filtering (cutting off the frequency from the top to specific values) and a selective or a band-based filtering. These filters are used to remove the noise from the signal, from frequencies outside the ranges of interest [7,13,14].

### 1.2. Criteria for the Selection of Transducers

In order to select the appropriate transducer, a task, which needs to be performed, must first be determined. The most important thing is to define the nature of the measurement by specifying the basic parameters, such as the type of measurement characteristic, the value of its magnitude, the accuracy, the way the result should be read and how often the reading will be taken. Due to this, by considering the advantages and disadvantages of a given solution, the appropriate equipment can be allocated. In addition, the designer of the measurement systems must meet certain criteria in order to select the optimal transducer. The costs of purchasing the apparatus, the performing measurements and maintenance, as well as the measuring accuracy, etc., could represent such criteria. It is equally important to consider whether the measuring device may have an influence on the tested system at the time of taking the measurements. The measurement of a liquid flow rate can be realized in many ways, e.g., by using solutions that require moving a rotor, which affect the liquid momentum; we talk about the so-called invasive method. However, when using, e.g., a magnetic-inductive meter, the use of the energy of the liquid for the purpose of conducting measurements is omitted, i.e., a non-invasive method is used. Moreover, it should be remembered that such devices only work for electrically conductive substances. It should be noted, from an engineering point of view, that all these criteria are often of secondary importance with regards to the basic question that is asked when selecting the optimal measuring device with a transducer: whether the lowest possible measurement error should be obtained, or whether the measurement should be obtained as soon as possible. This is due to the fact that it is difficult to achieve high measurement accuracy in a short time. Additionally, contrary to the error minimization criterion, the measurement speed is only important in systems with a high frequency of changes in parameters, or in automation and fault-tolerant control systems [8,9,10,11,15,16].

### 1.3. Optimization of Transducers

Like any system, the transducers can be optimized in order to improve the obtained results, which in this case means the reduction in the measurement error. The optimization is conducted for two purposes: for the tracking operations, and for processing the non-periodic signals [17].

In the case of the tracking operation, it is nearly impossible to conduct this task perfectly. This is due to the fact that a real transducer always shifts the signal phase, which causes delays in the measuring system. However, not every transducer in a specific system determines the dynamic properties of the measurement. It may happen that in one case the transducer is a non-inertial, i.e., it does not affect the measurement, while in another, it may significantly disturb the measurement. For example, the use of the transducer for static measurements is to perform measurements with a different and faster range of the velocity of the changes in signals [8].

A tracking transducer is usually modeled with an oscillating system of the second order with the following characteristic equation:

(6) $$\omega_{0}^{2}\frac{d^{2}x\left(t\right)}{dt^{2}}+2\omega_{0}\xi \frac{dx\left(t\right)}{dt}+x\left(t\right)=Ky\left(t\right)$$

where: *ξ* is the damping factor and *ω*_0_ is the period of undamped vibrations.

The damping factor ranges from 0 to 1. The model of the second-order inertial system is not used in this case because it causes too big a shift in the time of the signal. For an oscillating transducer, small damping values cause a small phase shift, i.e., the difference between the period of the oscillating signal and its response. Therefore, the appropriate selection of the damping allows the dynamic error to be reduced. In the case of optimization, due to the processing of non-periodic signals, an appropriate optimization criterion should also be adopted. This can be explained on the basis of a first-order inertial system, with the assumption that the unit jump test input signal [11] will be used.

### 1.4. Digital Meters

The digital meters function by processing a continuous voltage signal (usually) into a discretized form in a digital code [18,19]. It involves the sampling of the signal (i.e., the collecting of several values of a measured quantity within a specified period of time), which is then followed by its quantizing, assigning to each sample its corresponding number from a fixed finite set. Finally, the numbers are encoded as a series of impulses in a binary or a binary-coded decimal, which combines the features of the binary and the decimal notations in the binary and the decimal systems. These tasks are performed by AC transducers.

The digital meters are most often used to measure DC and AC voltage, frequency, capacity or resistance. They can be divided into two types, non-programmable and programmable, in other words, microprocessor-based. The latter are more interesting because, thanks to the above-mentioned microprocessors, they enable many measurement functions to be performed simultaneously. Due to the complexity of the meter implementation, the wording of the measurement systems should be used because appropriate requests can be sent on an ongoing basis via external keyboards or touch screens. The user sets the program that measures the parameter and the work regime, saving the results of the experiment or performing algebraic or trigonometric calculations [15,20].

In industrial metrology, the measurement of pressure is one of the most frequently performed measurements because it is a parameter of many technological processes. Due to the fact that industrial scale measurements are carried out at atmospheric pressure, the most common result is the relative pressure, and in rare cases, absolute pressure (the sum of the relative pressure and the atmospheric pressure). Overpressure is measured using manometers, negative pressure using vacuum gauges, pressure difference with the use of differential manometers and absolute pressure using barometers [14,21]. Apart from the pressure, the temperature measurement is also a very important parameter of the process that is crucial in many industries. The International Temperature Scale (ITS-90), which is an approximation of the thermodynamic absolute scale, is currently in force in the world.

This paper deals with the subject of generally understood industrial metrology. It describes the issues that are the basis for its practical part: the preparation of the measuring system, which is equipped with a total of six meters that are connected with the programmable logic controller. The creation of the measuring system to simultaneously monitor many parameters in an optimal and ergonomic way will allow the process quantities in question to be monitored. In practice, the system for monitoring the temperature, pressure and flow rate of fluids was constructed. It uses the operational and IT properties of the PLC controllers [22], and is adapted to the existing installation on the test stand for multiphase spraying processes. Such a system facilitates and accelerates scientific research that is related to the multiphase flows, and allows for the recording of the experimental data directly on a personal computer thanks to an implemented application written in Python. Such an application enables the collected data to be displayed, stored and exported. The entire system is used in a laboratory of the Faculty of Chemical Technology of Poznan University of Technology for the optimal simultaneous measurement of the temperature, pressure and flow rate in the flow processes under study [23].

## 2. Materials and Methods

This work aimed to design and create the measuring system equipped with 6 m connected to the PLC and to implement an application that allows displaying, collecting and exporting the collected measurement data. The huge variety of liquids or gases that are measured means that one universal solution should not be used. The selection of the correct flow/temperature/pressure meter should take into account the most important factors and parameters, including the type of liquid or gas to be measured, the diameter of the pipeline through which the medium will flow and even the requirements for the accuracy of the measurement. The proposed system is a simple, cheap and accurate solution that can, for example, support the process of educating students in the field of fluid mechanics, chemical engineering and measurement automation. The individual elements of the measuring system were selected on the basis of the measuring ranges of temperature, flow rate and pressure, which we deal with when spraying liquids with the use of two-phase atomizers. All elements were mounted on the test and the measurement installation on the supply lines (gas and liquid) of the tested two-phase atomizer. 

**Figure 1:**
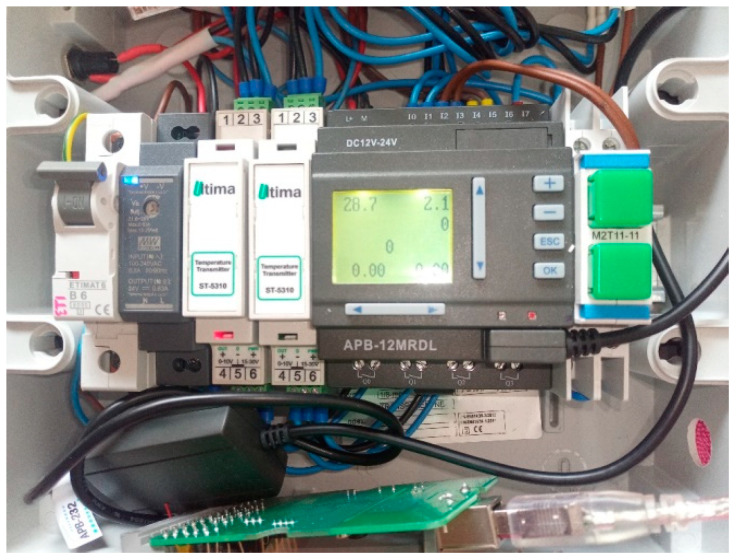
The PLC during operation. From the left: the circuit breaker, the power supply, the temperature transducers, the PLC, optional switch; below: the PLC–PC interface.

The measuring system was constructed, which is designed to measure many process quantities on a test stand based on the PLC unit (Figure 1), and connected with 6 sensors measuring liquid flow rate, pressure and temperature. Everything is powered using the 24 V DC power supply secured with a B6 circuit breaker. Due to the different internal structures and the identical inputs, the temperature sensors are protected by a fuse. In order to properly monitor the variable factors in the system, the application in Python programming language was developed. In addition, the application allows the measurements to be saved in the CSV format, which in turn makes the processing of data more convenient. During the saving of the measurement, the application displays the measurements in the form of a graph, which facilitates the optical determination of the condition of the measured system. The flow meters are connected to the PLC’s digital input because the sent signal is in the form of the impulses, indicating the number of the rotations of the turbine, which are moved by specified volume flowing through. The temperature and the pressure meters, on the other hand, send the analog signals, the measurement is converted by the built-in transmitters (in the case of the pressure meters) or the additional transmitters (the temperature sensors) to measure the voltage of the signal in range from 0 to 10 V.

**Figure 2:**
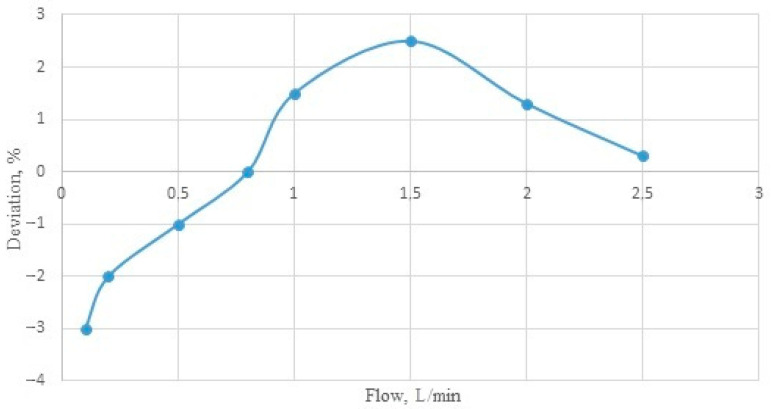
Diagram of the deviation in the measurement results for the flow sensor.

The FT-210 Gems Sensors (a flow turbine sensor) allow for the accurate testing of the liquid flow, regardless of its physical parameters, such as density or temperature. Each rotation of the rotor means that a certain amount of the liquid flows through it. For the FT-210 sensor, the minimum tested flow is equal to 1.7 mL/s, and the maximum is 41 mL/s. Within the measuring range, each revolution of the rotor generates an impulse, and 22 of these impulses represent a flow of 1 mL of the liquid. The accuracy of the meter is ±3% (Figure 2), and its repeatability is 0.5%. The flow sensors were calibrated before starting the tests. 

**Figure 3:**
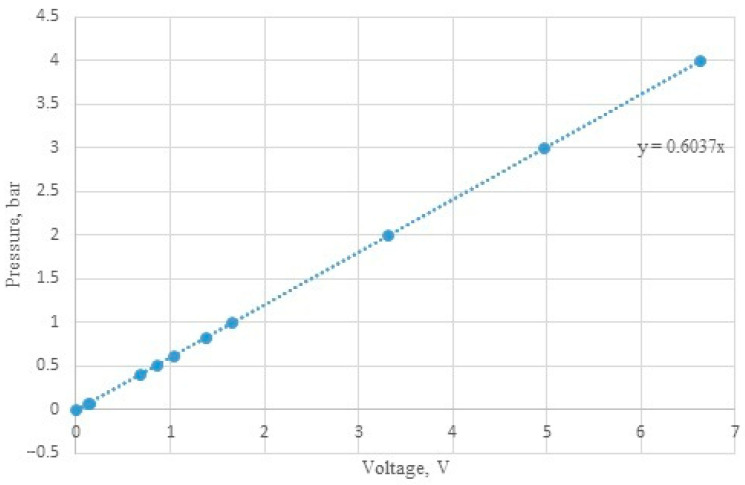
Calibration curve of the pressure sensor.

A piezoresistive Loxone pressure sensor with range 0–10 V was used. It measures the relative pressure with a repeatability of 0.5%, and within the range of 0–6 bar. As a result of the technological limitation of the PLC, it was necessary to introduce an appropriate calibration correction (Figure 3). Due to this, the measurement error was reduced to the lowest possible value. The pressure of the liquid (up to 2 bar) and the air (up to 4 bar) was verified using the Digicomb pressure sensor from Tecsis Company, and the obtained results were then compared with the readings of the system.

The device uses the TOPE-375 ACSE temperature sensor with the STU-5310-PT Ultima transducer. The PT100 resistance temperature sensor, with a range from −50 to 180 °C and a class B accuracy, is connected to the STU-5310-PT temperature transmitter, which in turn converts information from the temperature sensor into a voltage signal with a resolution of 0–10 V. Due to the use of the converter, the measuring range is 0–50 °C. The transmitters were calibrated with regards to a mixture of ice and water, which was also checked by a thermocouple connected to the Center 309 m from Center Company. 

### 2.1. Electrical Diagram of the System

**Figure 4:**
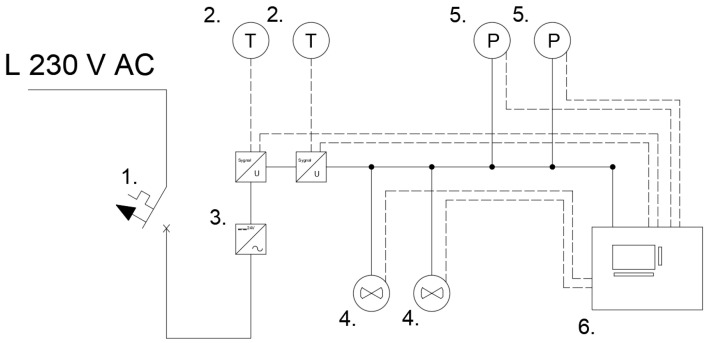
Diagram of the measuring system: (1) the overcurrent circuit breaker, (2) the temperature sensors with the signal/voltage transducers, (3) the DC power supply, (4) the flow sensors, (5) the pressure sensors, (6) the PLC unit.

The electrical diagram of the entire measuring system is presented in Figure 4. The definition of symbols is as follows: (1) the B6 circuit breaker, (2) the temperature sensors with the signal/voltage transducers, (3) the DC power supply, (4) the flow sensors, (5) the pressure sensors, (6) the PLC unit.

### 2.2. Scheme of Using the System

**Figure 5:**
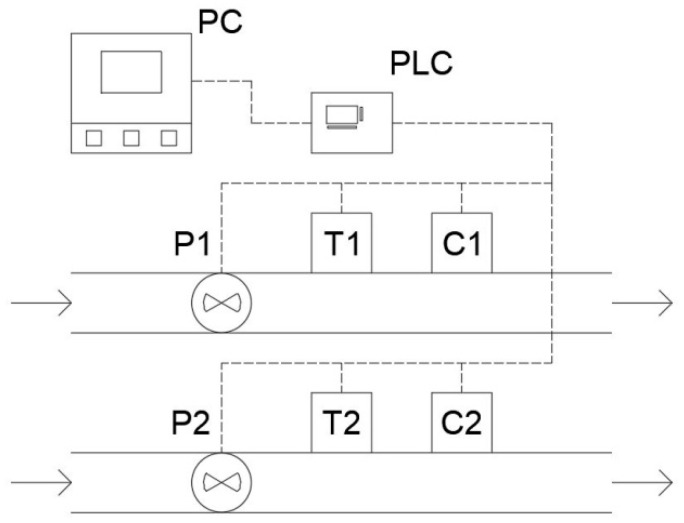
Scheme of the operating measuring system.

Figure 5 shows the schematic view of an example of the working system. Thanks to two sets of the sensors, it is possible to measure parallel objects, input and output from a reactor, or any other combination of the two systems. The PLC, which controls the sensors, collects the data that are downloaded every second by the application running on a personal computer.

### 2.3. The Monitoring Application

In order to ergonomically monitor the measurement, the desktop application was written in Python [23]. The connection of a personal computer with the PLC unit via the RS232/USB interface enabled data to be exchanged between these devices, which communicate using the Modbus protocol. The application downloads the data of the parameters, which are stored in the PLC registers, and then displays them in the appropriate GUI fields (Graphical User Interface). At the request of the user, the application can save measurements from the selected sensors and for the selected time period. In order to optimize the use of a computer memory, the files are saved every hour (maximum). During the saving of the measurements, the application displays the signal vs. the time graphs, which enables the measured system to be easily and quickly described.

**Figure 6:**
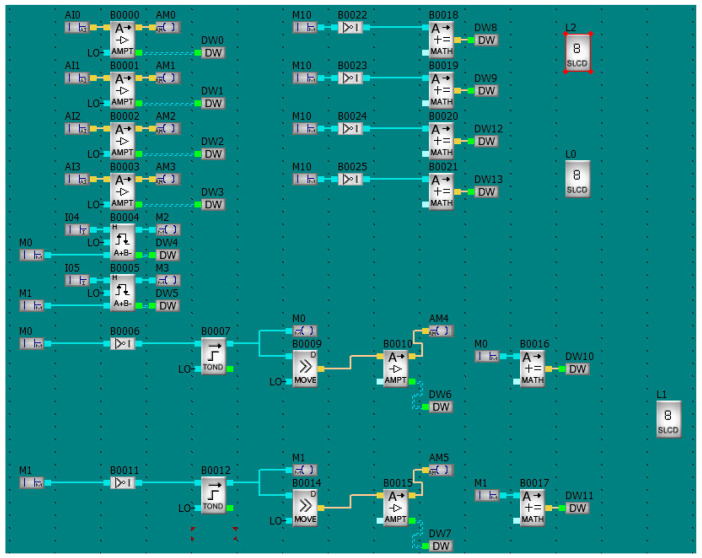
The PLC Program.

Figure 6 shows the program that supports the PLC, and, through the PLC, it also supports the sensors. The signal amplifications or the mathematical operations are carried out by the controller in order to avoid possible delays in the processing of the results during their display on the personal computer. The desktop application downloads the signal processing results from the prepared DW8–DW13 registers.

All the physical and logical inputs and outputs, as well as the individual program function blocks, have a hexadecimal code assigned to them. Therefore, the application can check the state of any object by sending a request to the controller’s processor.

**Figure 7:**
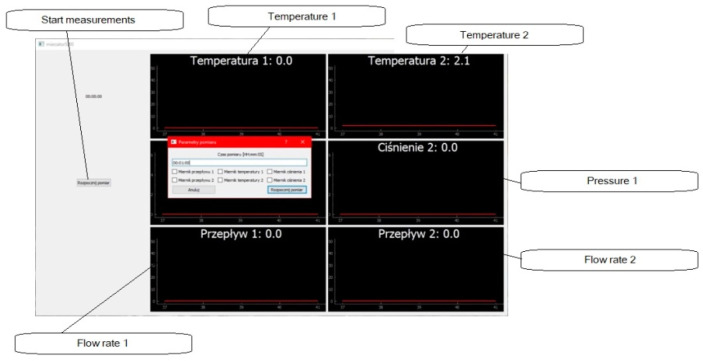
The main window of the measurement application with the “Measurement parameters” window (in Polish).

The operation of the measurement application (PC) can be divided into the three most important functions: creating an interface with charts (Figure 7), reading the measurements over time and saving them in the CSV format.

In the case of the first function, the code calls up the interface that includes a measurement start button, a measurement duration counter and six graphs, each corresponding to one of the sensors. After pressing the “Start measurement” button (Figure 7), another dialog window appears, which allows the sensor of interest and the duration of the recording of the measurements to be selected. After starting the measurement, the user can stop the measurement in progress, with the measurement still being saved.

**Figure 8:**
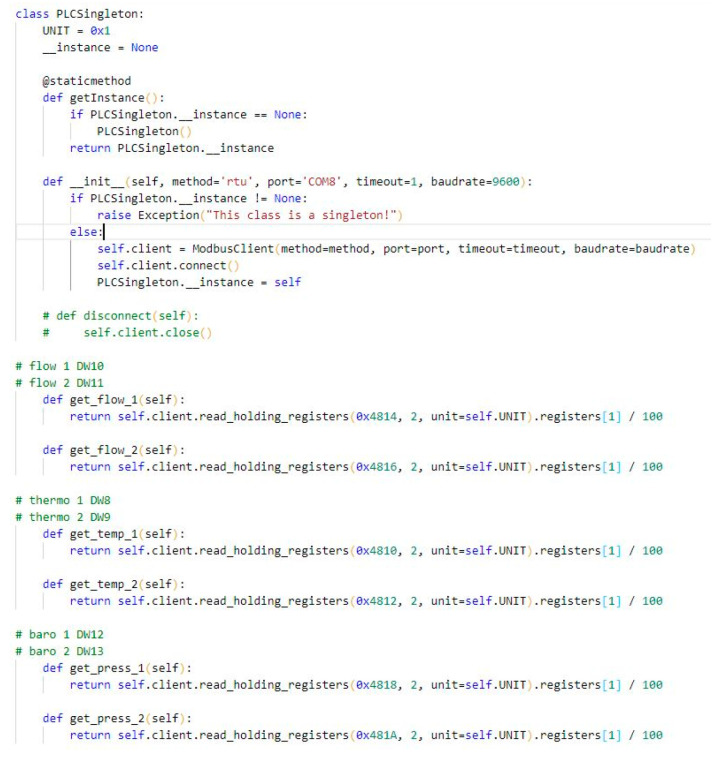
Functions that read the state of the PLC registers.

In order to monitor the parameters, the application needs to obtain information from the sensors. Therefore, by connecting the PLC to the personal computer, it is possible to read the values that appear in the registers. These data are distributed throughout the entire code wherever it is necessary (Figure 8).

**Figure 9:**
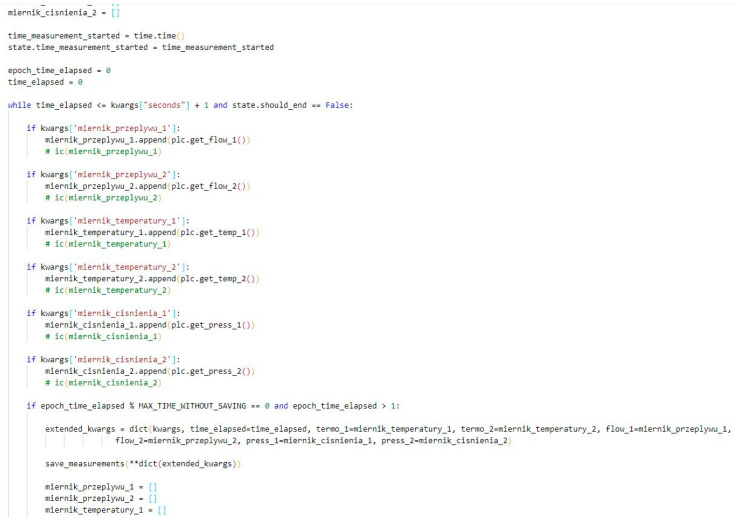
Fragment of the code that is responsible for recording the measurements.

The last function of the application involves the saving of the taken measurements. By default, the measuring system is in the passive monitoring mode: it shows the last 5 s of each sensor. However, when the user starts recording a measurement, the data are saved to the table every second (Figure 9). After completing the measurement, the tables are properly described and formatted in order for a file, which is easy to edit, to be obtained.

**Figure 10:**
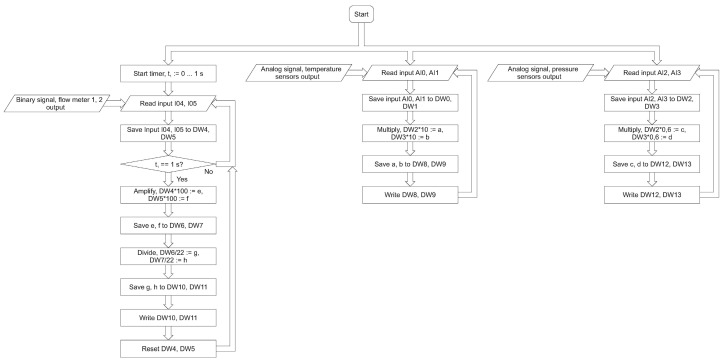
The flow meter algorithm.

One of the main tasks was the integration of the PLC with the desktop application. It requires a new algorithm, where the measurements’ accuracy will be consistent with the frequency of collection and with the transfer of the samples. The first algorithm is dedicated to the flow meter (Figure 10). It has three main loops dedicated to the reading of the analog sensors and the data persistence. This guarantees the possibility of the adaptation of or change in the sensor types without affecting the PC desktop application. The hardware part of the flow meter is separated from the user application; therefore, it can be integrated as part of a larger industrial system. The developed prototype has two uses. It is a new and precise research apparatus important from the point of view of further research. There is also a proof of concept in the field of hardware and software solution configuration in the context of the industrial implementation. In addition, the use of multithreading in the field of the sensor readings ensures greater accuracy of the measurements and correlation of the temperature and the pressure readings.

**Figure 11:**
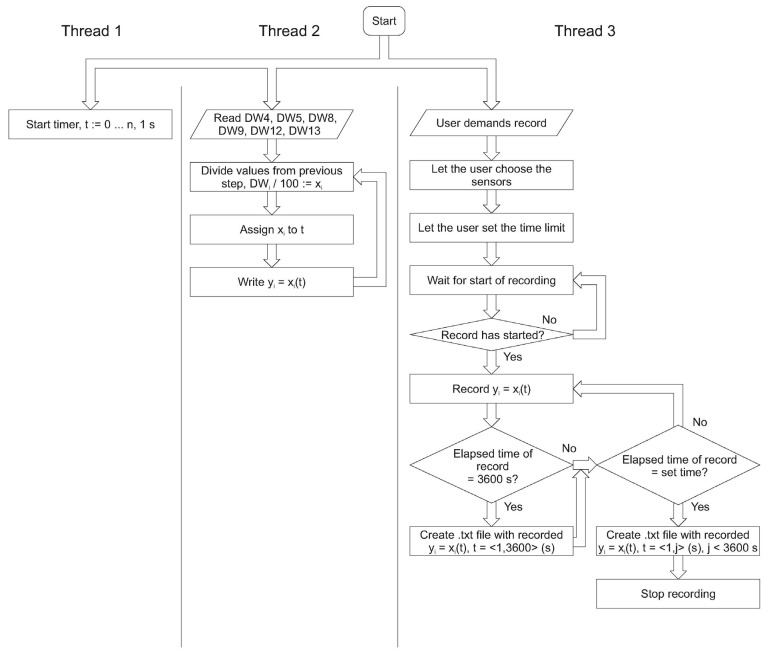
The desktop application algorithms.

Both in the scope of firmware for the PLC (Figure 10) and the desktop application (Figure 11), constant frequency of pressure and temperature readings was established for both sets of the sensors. The user application is multithreaded. The processes of reading the data, generating the graphs and recording the measurements are separated from each other. Probably thanks to this, the registration or the chart generation operations do not affect each other. The proposed solution enables the integration of the solutions in the field of the Internet of Things. The direction of further research should be the development of the IoT Gateway with the use of the COTS (commercial off-the-shelf) components. The advantage of this approach is the possibility of using (modernizing) older elements of the industrial lines. 

### 2.4. Measuring System

**Figure 12:**
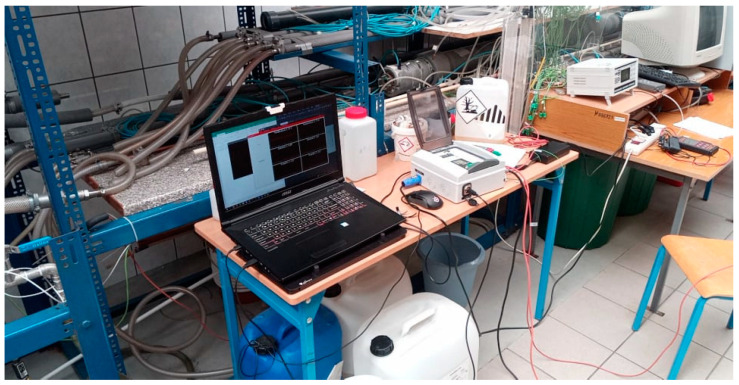
The PLC connected to the personal computer during the operation.

Figure 12 shows the assembled measuring system with the connected sensors, plugged into the PC computer. The screen shows the program window with the black measurement windows that present the current status of the measured values of temperatures, pressures and flow rates of the two working media.

The assembled system enables the selected quantity, which is displayed on the controller screen, to be read in real time. This function is useful when we do not want to connect the system to a PC, or when we want to record a single measurement. Currently, the above measuring system is installed on the multiphase flow test stand (multiphase spraying), on which the first tests have already been carried out in order to confirm its usefulness and efficiency during the simultaneous measurements of many quantities. The installed device will save time due to the automatic registration of the measurement series, the simultaneous measurement of several quantities, the saving of data in the form of a file that can be exported to MS Office Excel and the lack of the rotameter scaling [23,24,25].

**Figure 13:**
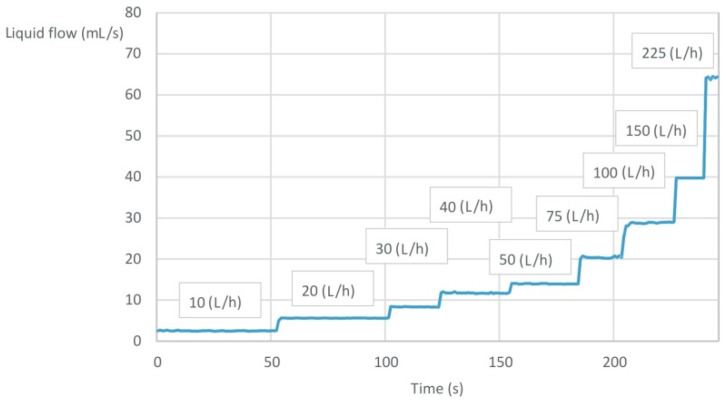
The exemplary dependence of the liquid flow rate values read on the rotameters (boxes) and the PLC meter (curve) on the time.

In order to validate the measurements, the results of the meter–controller system were compared with the results obtained from the Krohne VA 40 high accuracy rotameter battery (Figure 13). Numerous types of rotameters have been developed for different requirements. The rotameter accuracy varies depending upon the type of meter, scale length and calibration procedure. The rotameter is typically designed for one specific gas density and flow range, so without the rotameter being calibrated to those conditions, the accuracy will suffer. Calibrating the rotameter identifies the reference conditions in which the rotameter will be used, thus offering the most precise measurements. The uncertainty of the VA 40 is ±1.0% (acc. to VDI/VDE 3513, Sh. 2). In conformity with VDI/VDE 3513, Sh. 2, the accuracy for the variable area flow meters is defined by various accuracy classes. As for the uncertainty of the measurements, the accuracy is at the level of ±3% of the measurement.

**Figure 14:**
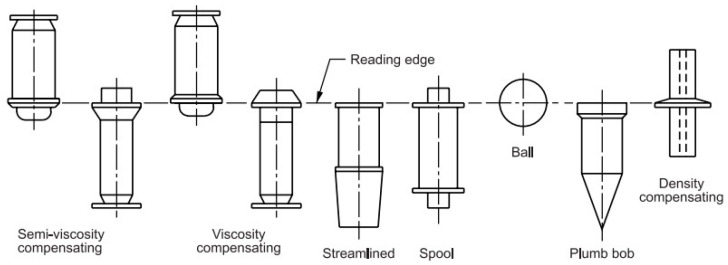
The exemplary types of floats.

The accuracy of the rotameters is affected by the changes in the fluid temperature. Judging float position can also be a problem because it depends on the skill of the reader and how steady the flow is. In practice, there are many types of floats (Figure 14). The actual error is therefore relatively difficult to assess. A poorly installed rotameter may suffer from an unsteady flow. The designed PLC meter does not have such disadvantages. In Table 1, exemplary comparisons of the flow rate values read on the rotameters and the registered data from the PLC meter are collected. 

**Figure 15:**
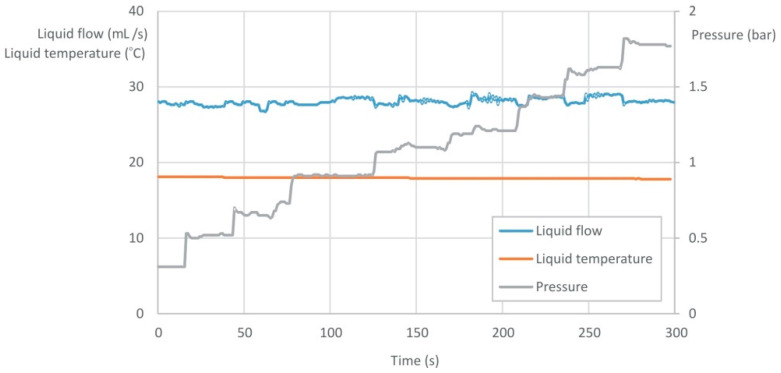
The exemplary dependence of liquid flow rate, liquid temperature and pressure on time for the different gas flow conditions (pressure rising).

Figure 15 shows an exemplary long-term record of the selected quantities for the two-phase gas–liquid flow in an effervescent atomizer. The liquid flow rate was set on constant (flow rate value for VA 40; 100 L/h), while the gas flow rate was changed. The values recorded and presented in Figure 15 were: liquid flow rate, pressure drop at the atomizer and liquid temperature. The average value of the liquid flow rate read by the PLC meter was 101 L/h with the accuracy ±3%.

The proposed solution is characterized by a direct reading of the flow rate, the pressure and the temperature, and then compared to the rotameters. It does not require calibration when changing the liquid and can record data online. Additionally, there are no errors related to the relative reading of the position of a float as is seen with the rotameters. The accuracy of the measurements is more than satisfactory and, generally, does not exceed ±3%.

## 3. Conclusions

The original assumption of the work was to create a measuring system, thanks to which the flow processes can be tested in an ergonomic and a simple way. For this purpose, the PLC was prepared to control the flow, pressure and temperature sensors. After preliminary tests, the sensors were calibrated in order to enable accurate and reliable measurements to be obtained. The data from the controller were then downloaded to the personal computer using the dedicated application. The application was created in such a way as to minimize the load on the PC, but at the same time to not distort the measurements, especially the time of their execution. As a result of research work, the integration algorithms were developed both on the side of the PLC and the user application. The solution can also be integrated into a larger industrial process. The conducted tests showed that the accuracy of the measurements reaches ± 3%, which is a satisfactory result.

The basic test was to connect all the sensors and then record their operation over the course of one hour, which is the maximum time for a single saved file. There were no problems found with the application, the PLCs or the sensors. It should be stated that the prepared system and the application implemented in Python meet all the set requirements, and that they were optimized to facilitate the scientific work of the staff. The installed device will allow time to be saved due to the automatic registration of the measurement series, the simultaneous measurement of several quantities, saving of the data in the form of a file and also the lack of the rotameter scaling.

## Figures and Tables

**Table 1:** The exemplary comparison of the flow rate values read on the rotameters and the PLC meter.

Flow Meter Type	Liquid Flow Rate (L/h)								
Rotameters VA 40	10	20	30	40	50	75	100	150	225
PLC meter	9.0 ± 1.0	20 ± 0.3	30 ± 0.2	41 ± 0.5	50.7 ± 0.4	73 ± 0.7	102 ± 1.6	143 ± 1.3	227 ± 2.2

## Data Availability

The data presented in this study are available in the article.
